# Dr. A. F. Helwig

**Published:** 1891-06

**Authors:** 


DR. A. F. HELWIG, of Swormville, Erie county, N. Y., died recently at his home. The Medical Society of Erie County has taken appropriate action, which will be published in the proceedings of the special meeting next month.

